# Supplementary material for: Coping strategies adapted by Ghanaians during the COVID-19 crisis and lockdown: A population-based study
Source: PLoS One. 2021 Jun 28;16(6):e0253800. doi: 10.1371/journal.pone.0253800 (PMC8238213; doi:10.1371/journal.pone.0253800)
Supplement: S1 Appendix — (PDF) [file pone.0253800.s001.pdf]

The questionnaire aspects used for the manuscript on "Coping Strategies Adapted by Ghanaians during the COVID-19 Crisis and Lockdown; a Population-based Study"

Have you read the information about this study and you agree to participate?

1. Yes
2. No

Welcome and thank you for taking part in this survey. The information you will share with us will contribute to a scientific study, so kindly answer as truthfully as possible.

Have you participated in this study before?

1. First time
2. 2.Second time
3. 3.Third time
4. 4.Forth time
5. 5.Fith time
6. 6.Sixth time
7. 7.Seventh time

Section 1

In what country do you live?

Do you live in an urban or rural area?

1.Urban

2.Rural

3.prefer not to say

What is your sex?

1 = Female

2 = Male

3 = Prefer not to say

What is your age range?

1 = Under 18 years old.

2 = 18-24years old.

3 = 25-34years old.

4 = 35-44years old.

5 =45-54 years old.

6 = 55-64 years old

7=65 +

8=prefer not to say/dont know

Which of the following best describes your current relationship status?

1 = Married

2 = widowed

3 = Separated/Divorced

4 = single but cohabiting with a significant other

5 = Single/never married

6. Prefer not to say/I dont know

How many children do you have?

1= No children

2= one

3= Two

4= Three

5 = Four

6 = More than four

7=prefer not to say/dont know

What is you highest level of education?

1= I didn't attend school

2=Primary

3= Secondary

4= Bachelor

5=Masters

6=Doctorate/PhD

7=prefer not to say/dont know

What is your work situation?

1= no employment

2= agriculture/farming

3= daily worker/small income job

4= employee

5= government employee

6= business owner

7=prefer not to say

In the context of your country, what economic category fits best with your situation?

1: low income

2: lower middle income

3: higher middle income

4: high income

Section 2

1.Is the country where you live implementing the lockdown measures?

1=Yes

2=No

3=Not sure

2.Have you yourself been infected by the COVID-19 virus?

1=Yes

2=No

3=Not sure

3.Has someone in your household been infected by the COVID-19 virus?

1=Yes

2=No

3=Not sure

4.Has someone close to you been infected by the COVID-19 virus?

1=Yes

2=No

3=Not sure

5.Has someone close to you died from the COVID-19 virus?

1=Yes

2=No

3=Not sure

6.How concerned are you about your own health and the health of your family in relation to the COVID-19 virus?

Not at all concerned (5)

Slightly concerned (4)

Somewhat concerned (3)

Moderately concerned (2)

Extremely concerned (1)

7.Are you concerned about supporting your family financially because of the COVID-19 virus?

1=Yes

2=No

3=Not sure

8.How difficult do you find it to switch off from media (TV news, newspapers) and social media (Twitter, Whatsapp) reporting about COVID-19?

Very easy (1)

Easy(2)

Neither easy nor difficult (3)

Difficult(4)

Very difficult(5)

9.Weighing the positives and the negatives, has your life become better or worse since the COVID-19 crisis started?

Much worse (5)

worse (4)

About the same (3)

Better(2)

Much better(1)

10. Do you think the situation in your country will be well controlled or will it go out of control?

Very well controlled (1)

Somewhat controlled (2)

Neutral (3)

Out of control (4)

Totally out of control (5)

11. We will present to you a number of activities that you typically do at home. Kindly indicate whether in this period you engage in them less, the same or more compared to before the lockdown

a) Watching tv

Less (1) Same (2) More (3) Prefer not to say/Not applicable (4)

b) Spending time on internet (not for work)

Less (1) Same (2) More (3) Prefer not to say/Not applicable (4)

c) Spending time on social media (not for work)

Less (1) Same (2) More (3) Prefer not to say/Not applicable (4)

d) Working from home or performing other income generating activities

Less (1) Same (2) More (3) Prefer not to say/Not applicable (4)

e) Performing household chores; doing house repairs

Less (1) Same (2) More (3) Prefer not to say/Not applicable (4)

f) Doing sports

Less (1)   Same (2)   More (3)   Prefer not to say/Not applicable (4)

g) Talking to friends and relatives over the phone

Less (1)   Same (2)   More (3)   Prefer not to say/Not applicable (4)

h) Praying

Less (1)   Same (2)   More (3)   Prefer not to say/Not applicable(4)

i) Resting/relaxing

Less (1)   Same (2)   More (3)   Prefer not to say/Not applicable (4)

j) Sleeping

Less (1)   Same (2)   More (3)   Prefer not to say/Not applicable (4)

In your own appreciation, do you think you are coping well with the COVID-19 situation?

1. Not well at all

2.Not well

3.Average

4.Well

5.Very well
