# Supplementary material for: Coping strategies adapted by Ghanaians during the COVID-19 crisis and lockdown: A population-based study
Source: PLoS One. 2021 Jun 28;16(6):e0253800. doi: 10.1371/journal.pone.0253800 (PMC8238213; doi:10.1371/journal.pone.0253800)
Supplement: Attachment — Submitted filename: Response to Reviewers.docx [file pone.0253800.s003.docx]

**Response to Reviewers**

| **Academic Editor** | **Responses** |
| --- | --- |
| Please include the following items when submitting your revised manuscript:   - A rebuttal letter that responds to each point raised by the academic editor and reviewer(s). You should upload this letter as a separate file labeled 'Response to Reviewers'. - A marked-up copy of your manuscript that highlights changes made to the original version. You should upload this as a separate file labeled 'Revised Manuscript with Track Changes'. - An unmarked version of your revised paper without tracked changes. You should upload this as a separate file labeled 'Manuscript'. | We thank the editor for the opportunity to revise the manuscript for consideration.  We have considered all the comments from the Editor and the reviewer and have provided a point-by-point response to the comments.  Both the ‘revised manuscript with track changes’ and ‘the unmarked version of the manuscript’ are uploaded as separate files. |
| **Journal Requirements/Comments** |  |
| 1. Please provide additional details regarding participant consent. In the ethics statement in the Methods and online submission information, please ensure that you have specified what type you obtained (for instance, written or verbal, and if verbal, how it was documented and witnessed). If your study included minors, state whether you obtained consent from parents or guardians. If the need for consent was waived by the ethics committee, please include this information. | The online survey tool begins with a section providing participant’s information about the study and then end with the question “have you read the information about this study and you agree to participate?” with a ‘yes’ and ‘no’ response. Only those who selected ‘yes’ have the questionnaire opened for them to participate. This is indicated on lines 99-100.  We stated in the section “**Data collection and ethical clearance**” that “Participation was voluntary and participants could skip questions they did not wish to respond to. The online form required the participant to read the background information on the study and then select to indicate if they were ready to participate before they were able to proceed to respond to the questions.”  Also, The minimum age for participating was 18 years. This was stated on line 81 as follows:  “The study population included participants aged 18 years or older, the ability to provide informed consent, and residents in partner countries, and in the case of this paper, residents in Ghana.” |
| 2. During our internal checks, the in-house editorial staff noted that you conducted research or obtained samples in another country. Please check the relevant national regulations and laws applying to foreign researchers and state whether you obtained the required permits and approvals. Please address this in your ethics statement in both the manuscript and submission information. | We thank the reviewer for pointing out this confusion. Please note that this paper reports the part of the multi-country study conducted in Ghana. Thus we did not conduct this research or obtain the data from another country but Ghana. The consortium worked in such a way that each country team obtained ethical clearance to collect data in that country. So the data presented here represent only research conducted/data obtained in Ghana.  We have clarified this in the manuscript with the following statement (line 83 and 91).  “This paper utilizes data obtained from Ghana. Participants residing in Ghana were recruited from different social media platforms and personal contact via phone calls. |
| 3. During the internal evaluation of the manuscript we have noted that the current study is a part of a a multi-country online cross-sectional survey on Personal and Family Coping with COVID-19. Please provide a citation for this study. | We are grateful for this comment. We wish to state that there is presently no citation for this study. Each country team is in the process of finalizing country-level analysis and manuscript preparations and submissions for publication. |
| Please include additional information regarding the survey or questionnaire used in the study and ensure that you have provided sufficient details that others could replicate the analyses. For instance, if you developed a questionnaire as part of this study and it is not under a copyright more restrictive than CC-BY, please include a copy, in both the original language and English, as Supporting Information. | Thank you for the comment. We have included a copy of the questionnaire which contains information used for this manuscript as a suplementary file Appendix 1: S1 Questionnaire.pdf. |
| Please include as Supporting File a list of all participating countries | The countries which participated and got the minimum agreed number of participants were Uganda, Bangladesh, Rwanda, Indonesia, Ghana, Brazil, Myanmar (Burma), Malaysia, Cameroon, and Kenya. But kindly note that this paper is based on only the Ghana study site.  We have captured this in the manuscript with the following sentence.  “The larger study was designed to represent populations in 10 countries (Uganda, Bangladesh, Rwanda, Indonesia, Ghana, Brazil, Myanmar (Burma), Malaysia, Cameroon, and Kenya).” |
| 4. We note that you have indicated that data from this study are available upon request. PLOS only allows data to be available upon request if there are legal or ethical restrictions on sharing data publicly. For information on unacceptable data access restrictions, please see <http://journals.plos.org/plosone/s/data-availability#loc-unacceptable-data-access-restrictions>. | The dataset used in this paper is provided as a supporting information file. We have added the following sentence to the manuscript:  “The dataset used in this paper is provided as a supporting information file (see Appendix 2: S2 Dataset.xlsx).” |
| **Reviewer #1** |  |
| *Abstract:* 1. Line 30: Authors may revise the sentence to “This study was..” by adding “study” | Corrected. Thank you. See line 30 |
| *Materials and Methods* 2. Line 82-83: “Participants were recruited from different social media platforms and personal contact via phone calls.” How can authors re-assure readers that a participant didn't complete the questionnaire more than once? Any safeguards? | The online questionnaire had a question to indicate if a participant is participating for the first, second, etc. time. The analyses presented in this paper are based on only the first responses. The dataset has been attached as a supplementary file.  See lines 84-86 |
| 3. Line 86: Is it “Cohen’s d” or “Cohen’s dz? | We have corrected this to Cohen’s *d*. Line 88 |
| 4. Line 95: “The online questionnaire is composed of different psychometrically validated instruments”. Can authors report the reliability and validity values for these scales among Ghanaian samples? | Thank you for the comment. We have included a copy of the questionnaire which contains information used for this manuscript as a suplementary file Appendix S1. And modified that portion in the manuscript.  Lines 97-101 |
| *Results* 5. Lines 179-190: Especially the first sentence (ordered logistic regression are shown in Table 6) seems to be misleading as it did not reflect in Table 6. Hence, authors may re-look at it and revise it appropriately. | We have corrected this. The statement was referring to Table 7. |
| 6. Lines 193-206: Authors seem to report data (figures) that cannot be verified from its respective Table (Table 7). Also, there were typos (e.g., line 201). | We thank the reviewer for pointing these inconsistencies out. We inadvertently presented table for a set of different analyses. We have corrected the figures in the Table. Please note that this did not affect the interpretation of the results. We have highlighted figures in the new Table 7 that had changes in the numbers. |
| *Discussion* 7. Line 229: Does Ghana belong to low-income country? | Ghana is classified as a lower-middle-income country. This has been clarifies. Line 233. |
| 8. Line 235-238: Please cite some studies to support the sentence. | Thank you. This has been done. Line 240. |
| 9. Line 244-245: “A total…” can authors use percentages in the sentence instead of absolute figures? | We have reported the percentages as requested. Lines 246-247. |
| 10. Lines 245-246: “These were found to be associated with decreased coping.” What are “these”? | The sentence does not add any further insight and has therefore been deleted. |
| 11. Lines 270-271: “…will reduce the impact of any economic impact the pandemic had on people.” Can authors replace of the impacts with another word? | Thanks. We have replaced ‘impact’ with ‘effect’. Line 272 |
| 12. Lines 276-277: “…relative to those with a pre-existing condition.” Is it “without” rather? | Correction made. Line 279 |
| 13. There are few grammatical and typos. Hence, authors may have to proof-read and revise appropriately. | Thank you and this has been done throughout the manuscript |
